# Meteorological Table

**Published:** 1812-12

**Authors:** 


### METEOROLOGICAL TABLE.

*From the 25th of October, to the 25th of November.*

| | D. | Therm. | | | Barom. | | Hygrom. Dry.——Damp. | | | | Winds. | Atmos. Variation. |
|---|---|---|---|---|---|---|---|---|---|---|---|---|
| | 26 | 47 | 52 | 45 | $29^5$ | $29^7$ | | 8 | 6 | 3 | W.. | C... F.. C.. |
| ◑ | 27 | 44 | 46 | 44 | $29^8$ | $^4$ | | 6 | 10 | 8 | W.... | C... R... F.. |
| | 28 | 42 | 45 | 42 | $29^4$ | $^6$ | | 8 | 4 | 6 | W.. | F.. |
| | 29 | 39 | 48 | 41 | $29^8$ | — | | 4 | 2 | 7 | W.. | F. |
| | 30 | 45 | 54 | 48 | — | $29^7$ | | 8 | 6 | 4 | S.. | C... R... |
| | 31 | 44 | 55 | 49 | $29^8$ | 30 | | 7 | 3 | 7 | S.. | F.. |
| | 1 | 53 | 54 | 52 | 30 | $29^9$ | | 7 | 5 | 7 | W | C.. R.. |
| | 2 | 53 | 56 | 51 | — | 30 | | 10 | 2 | 6 | NW | R... F... R... |
| | 3 | 48 | 50 | 44 | — | $30^1$ | | 9 | 6 | 9 | NW | C... |
| ● | 4 | 49 | 44 | 45 | 30 | — | | 10 | 1 | 3 | NW | C... |
| | 5 | 43 | 48 | 44 | $29^9$ | — | — 3 — | 1 | — | 1 | NW | F.. |
| | 6 | 31 | 41 | 40 | $29^8$ | $29^7$ | | 3 | 1 | 3 | N | F.. |
| | 7 | 35 | 40 | 38 | — | $29^8$ | | 4 | 3 | 5 | N | C... |
| | 8 | 34 | 37 | 38 | | | | 3 | 2 | 1 | E | F.. |
| | 9 | 40 | 42 | 39 | — | 30 | | 3 | — | 4 | E | C... |
| | 10 | 36 | 46 | 43 | $30^1$ | — | | 2 | 3 | 4 | S | C... |
| | 11 | 43 | 47 | 43 | — | $29^8$ | | 2 | 4 | 5 | SE | C.. |
| ◐ | 12 | 40 | 42 | 44 | — | $29^6$ | | 6 | 5 | 8 | E | R.. F. |
| | 13 | 48 | 51 | 49 | — | $29^3$ | | 8 | 10 | 12 | SE | C.. |
| | 14 | 52 | 55 | 50 | $29^6$ | — | | 7 | 10 | 11 | S | C.. |
| | 15 | 45 | 50 | 45 | $29^6$ | $29^5$ | | 10 | 8 | 10 | SW | F. |
| | 16 | 43 | 47 | 41 | $29^4$ | $29^2$ | | 4 | 7 | 10 | W | R.. C.. R.. |
| | 17 | 47 | 49 | 44 | 29 | — | | 9 | 10 | 10 | W | R.. |
| ○ | 18 | 44 | 47 | 40 | $29^2$ | $29^5$ | | 8 | 10 | 7 | N | R. F.. |
| | 19 | 33 | 40 | 37 | $29^7$ | $29^8$ | | 2 | 6 | 8 | N | F... |
| | 20 | 32 | 39 | 38 | $29^8$ | — | | 5 | 2 | 5 | E | C.. |
| | 21 | 37 | 38 | 36 | 30 | $30^2$ | | 2 | 5 | 6 | E | C.. |
| | 22 | 34 | 37 | 35 | $30^3$ | — | | 4 | 6 | 7 | E | F. |
| | 23 | 30 | 38 | 41 | — | $30^2$ | | 7 | 6 | 8 | E | Fog... |
| | 24 | 42 | 45 | 41 | $30^1$ | $29^9$ | | 7 | 8 | 9 | SW | F. |
| ◑ | 25 | 44 | 46 | 42 | $29^8$ | $29^7$ | | 10 | 7 | 5 | SW | C... |

Quantity of rain from October the 25th to November the 25th, two inches $\frac{35}{100}$.

The 20th severe frost. Ice an inch and a half thick in the country, at a short distance from London. Fogs have been frequent, and particularly dense on the morning of the 20th.

The case of supposed Typhus, mentioned in the preceding Table as in a state of convalescence, terminated fatally, after the subsidence of the fever, by a sudden hæmorrhage from the intestines. Small-pox has, and is, prevailing to a considerable extent, and the bills present, weekly, many instances of death by this disease. Two cases of variola after vaccination have occurred: one in London, and the other at Hackney. The last affords the singular fact of the patient being apparently secure from the contagion of small-pox in the 8th year after vaccination, but caught the disease in the ninth. Pulmonic affections are becoming frequent, and some cases of measles have appeared. Some children have also been affected with mumps.

MONTHLY

